# Apoptotic adipose-derived mesenchymal stem cell therapy protects against lung and kidney injury in sepsis syndrome caused by cecal ligation puncture in rats

**DOI:** 10.1186/scrt385

**Published:** 2013-12-25

**Authors:** Pei-Hsun Sung, Chia-Lo Chang, Tzu-Hsien Tsai, Li-Teh Chang, Steve Leu, Yung-Lung Chen, Chic-Chao Yang, Sarah Chua, Kuo-Ho Yeh, Han-Tan Chai, Hsueh-Wen Chang, Hong-Hwa Chen, Hon-Kan Yip

**Affiliations:** 1 Division of Cardiology, Department of Internal Medicine, Kaohsiung Chang Gung Memorial Hospital and Chang Gung University College of Medicine, Kaohsiung, Taiwan; 2 Division of Colorectal Surgery, Department of Surgery, Kaohsiung Chang Gung Memorial Hospital and Chang Gung University College of Medicine, Kaohsiung, Taiwan; 3 Basic Science, Nursing Department, Meiho University, Pingtung, Taiwan; 4 Center for Translational Research in Biomedical Sciences, Kaohsiung Chang Gung Memorial Hospital and Chang Gung University College of Medicine, Kaohsiung, Taiwan; 5 Division of Nephrology, Department of Internal Medicine, Kaohsiung Chang Gung Memorial Hospital and Chang Gung University College of Medicine, Kaohsiung, Taiwan; 6 Department of Biological Sciences, National Sun Yat-Sen University, Kaohsiung, Taiwan; 7 Institute of Shock Wave Medicine and Tissue Engineering, Kaohsiung Chang Gung Memorial Hospital and Chang Gung University College of Medicine, Kaohsiung, Taiwan

## Abstract

**Introduction:**

We tested the hypothesis that apoptotic adipose-derived mesenchymal stem cells (A-ADMSC) are superior to healthy (H)-ADMSC in attenuating cecal ligation puncture (CLP)-induced sepsis-mediated lung and kidney injuries.

**Methods:**

Adult male rats divided into group 1 (sham controls), group 2 (CLP), group 3 [CLP + H-ADMSC administered at 0.5, 6, and 18 hours after CLP], and group 4 [CLP + A-ADMSC administered as in group 3] were sacrificed 72 hours after CLP with blood, lung, and kidney collected for studies.

**Results:**

White blood cell (WBC) count, circulating TNF-α and creatinine levels were higher in groups 2 and 3 than in groups 1 and 4 (all P < 0.001). Kidney and lung damage scores were highest in group 2, lowest in group 1, significantly higher in group 3 than in group 4 (all P < 0.0001). Protein expressions of inflammatory (ICAM-1, MMP-9, TNF-α, NF-κB), oxidative, and apoptotic (Bax, caspase-3, PARP) biomarkers were higher in groups 2 and 3 than groups 1 and 4, whereas anti-apoptotic (Bcl-2) and mitochondrial integrity (cytochrome-C) biomarkers were lower in groups 2 and 3 than in groups 1 and 4 (all P < 0.001). Expressions of anti-oxidant biomarkers at protein (GR, GPx, NQO-1, HO-1) and cellular (GR, GPx) levels were highest in group 4 (all P < 0.001). The number of inflammatory cells (CD3+) in lungs and levels of DNA damage marker (γ-H2AX) in kidneys were higher in groups 2 and 3 than in groups 1 and 4 (all P < 0.001).

**Conclusions:**

A-ADMSC therapy was superior to H-ADMSC therapy in protecting major organs from damage in rats with CLP-induced sepsis syndrome.

## Introduction

Despite the state-of-the-art treatment strategies and advanced antibiotic regimens, acute sepsis syndrome, a global problem, remains the leading cause of death in hospitalized patients with infectious disease [1-4]. Death from sepsis syndrome is caused by a complicated process with the involvement of divergent factors [5-7]. Of these factors, overwhelming immune response (that is, host response) and inflammatory reaction [4-6] are major contributors to direct or indirect assaults on the vital organs [8-11]. In addition, studies have shown that sepsis syndrome-related acute lung injury (ALI) and acute kidney injury (AKI) are commonly encountered in critically ill patients [10]. Poor prognostic outcomes have been reported for patients in these clinical settings [6,9-15]. Therefore, in addition to the appropriate choice of antibiotics [1,2], the control of the overwhelming immune response and vigorous inflammatory reaction that contribute to the damage of lung and kidney, two vital and vulnerable organs, may be crucial in reducing sepsis-induced morbidity and mortality [5-7].

Not only has growing evidence demonstrated that stem cells possess the intrinsic capacity of immunomodulation [15-17], but stem cell therapy has also been shown to attenuate inflammation and immune responses [15-19] and augment wound healing cells to favor tissue regeneration and inhibit fibrotic tissue formation [19]. Additionally, utilizing three-dimensional culture models, stem cells have been shown to have the capacity of tissue formation and organ reconstruction [20]. Also, it has been proposed that apoptotic/dying stem cells exhibit distinctive immunosuppressive properties [21]. Moreover, our data recently showed that apoptotic adipose-derived mesenchymal stem cells (A-ADMSC) were superior to healthy ADMSC (H-ADMSC) in attenuating acute lung ischemia-reperfusion (IR) injury in rats through suppressing inflammation, oxidative stress, reactive oxygen species (ROS) generation and immune response [22].

Experimental studies have previously demonstrated that, in the setting of sepsis syndrome, stem cell treatment notably alleviated sepsis-induced inflammatory reaction, decreased mortality and improved prognostic outcome [23-25]. Additionally, in another recent study we showed that A-ADMSC therapy was superior to H-ADMSC therapy for preserving heart function and reducing mortality in rat sepsis syndrome [26]. Therefore, it is rational to believe that there is a potentially more important role for A-ADMSC therapy for sepsis syndrome. However, whether A-ADMSC is superior H-ADMSC in protecting the kidney and lung against sepsis syndrome-associated injuries is currently unclear. It is well recognized that appendicitis-induced peritonitis is one of the most common clinically encountered sepsis syndromes in our daily practice. Accordingly, using a rodent model of cecal ligation and puncture (CLP)-induced sepsis syndrome, this study tested the hypotheses that: (1) CLP-induced sepsis syndrome may significantly contribute to ALI and AKI; (2) ADMSC treatment may significantly attenuate sepsis-induced kidney and lung injuries; and (3) A-ADMSC may be superior to H-ADMSC in reducing sepsis syndrome-associated lung and kidney injury.

## Methods

### Ethics

All animal experimental procedures were approved by the Institute of Animal Care and Use Committee at our hospital and performed in accordance with the Guide for the Care and Use of Laboratory Animals (NIH publication No. 85–23, National Academy Press, Washington, DC, USA, revised 1996). All technicians who performed the bench work were blinded to the treatment protocol.

### Animal grouping and isolation of adipose tissue for culturing adipose-derived mesenchymal stem cells

Pathogen-free, adult male Sprague–Dawley (SD) rats weighing 350 to 400 g (Charles River Technology, BioLASCO Taiwan Co. Ltd., Yilan, Taiwan) were categorized into sham procedure (SC) (that is, cecal exposure without ligature and puncture), CLP only (CLP + saline 3.0 mL, intra-peritoneally at 30 minutes, 6 hours, and 18 hours after CLP), CLP + H-ADMSC ((autologous 1.2 × 10^6^ cells) at 30 minutes, 6 hours, and 18 hours after CLP), and CLP + A-ADMSC ((autologous 1.2 × 10^6^ cells) at 30 minutes, 6 hours, and 18 hours after CLP); n = 8 animals per group.

The choice of time points of ADMSCs administration at 30 minutes, 6 hours, and 18 hours after CLP was based on our recent study that demonstrated that ADMSC administration at these time points after acute rat kidney IR injury through penile venous transfusion markedly attenuated acute ischemia/reperfusion-induced kidney injury [16]. In addition, these time points were initially chosen in an attempt to mimic the clinical scheduling of antibiotics for patients with sepsis syndrome.

Rats in groups CLP + H-ADMSC and CLP + A-ADMSC were anesthetized with inhalational 2.0% isoflurane 14 days before CLP to harvest autologous peri-epididymal adipose tissue as we recently reported [16]. The procedure and protocol for culture and identification of H-ADMSCs and A-ADMSCs were described in our recent reports [16,18,22]. Briefly, the adipose tissue surrounding the epididymis was carefully dissected and excised. Then 200 to 300 μL of sterile saline was added to every 0.5 g of tissue to prevent dehydration. The tissue was cut into <1 mm^3^ size pieces using a pair of sharp, sterile surgical scissors. Sterile saline (37˚C) was added to the homogenized adipose tissue in a ratio of 3:1 (saline: adipose tissue) by volume, followed by the addition of stock collagenase solution to a final concentration of 0.5 Units/mL. The tubes with the contents were placed and secured on a Thermaline shaker and incubated with constant agitation for 60 ± 15 minutes at 37°C. After 40 minutes of incubation, the contents were triturated with a 25 mL pipette for 2 to 3 minutes. The cells obtained were placed back on the rocker for incubation. The contents of the flask were transferred to 50 mL tubes after digestion, followed by centrifugation at 600 g for 5 minutes at room temperature. The lipid layer and saline supernatant from the tube were poured out gently in one smooth motion or removed using vacuum suction. The cell pellet thus obtained was resuspended in 40 mL saline and then centrifuged again at 600 g for 5 minutes at room temperature. After being resuspended again in 5 mL saline, the cell suspension was filtered through a 100 μm filter into a 50 mL conical tube to which 2 mL of saline was added to rinse the remaining cells through the filter. The flow-through was pipetted to a 40 μm filter into a new 50 mL conical tube. The tubes were centrifuged for a third time at 600 g for 5 minutes at room temperature. The cells were resuspended in saline. Isolated ADMSCs were cultured in a 100 mm diameter dish with 10 mL (D)MEM culture medium containing 10% fetal bovine serum (FBS) for 14 days.

### The procedures of cecal ligation and puncture and measurement of tail systolic blood pressure

Rats were anesthetized with inhalational 2.0% isoflurane and placed in a supine position on a warming pad at 37°C with the abdomen shaved. Under sterile conditions, the abdominal skin and muscle were opened and the cecum exposed in all groups. In the sham controls, the abdomen was then closed and the animal was allowed to recover from anesthesia. In the experimental CLP groups, the cecum was prolene suture ligated over its distal portion (that is, distal ligation) and the cecum distal to the ligature was punctured twice with an 18G needle to allow the cecal contents to be expressed intraperitoneally, as previously described [27]. The abdominal wound was closed and the animal was allowed to recover from anesthesia.

The tail systolic blood pressure (SBP) was measured (Kent Scientific Corporation, Model no: CODA, Torrington, CT, USA.) by a technician who was blinded to the treatment protocols prior to and at 9 hours and 18 hours after CLP or the sham procedure. The tail cuff approach to blood pressure measurement was conducted as follows: initially the rat was warmed in a box at 37°C for 20 minutes before being placed in a restraining apparatus which was also kept at 37°C. The tail was inserted through the cuff which contained a photoelectric pulse detector through which the systolic blood pressure was recorded when the first oscillation appeared during the gradual reduction of cuff pressure. Mean blood pressure was determined from the cuff pressure when the amplitude of the oscillation reached its maximum. The SBP was consecutively and continuously measured 30 times in each rat. The value of SBP recorded for the first 10 times and those recorded when the animals were agitated were discarded. Finally, reliable SBP readings were averaged for each rat and expressed as mean ± SD.

### Definition of healthy and apoptotic ADMSCs

Healthy ADMSCs were those cultured in normal culture medium with adequate nutrition. Serum deprivation of cells *in vitro* induces apoptosis [22,28]. Hence, apoptotic ADMSCs were first cultured in normal culture medium followed by 96 hours of serum-free cell culture. The percentages of viable and apoptotic cells were determined by flow cytometry using double staining of annexin V and propidium iodide (PI); this is a simple and popular method for the identification of apoptotic cells (that is early (annexin V+/PI-) and late (annexin V+/PI+) phases of apoptosis).

The principal concept in the present study for the induction of A-ADMSCs using ‘serum deprivation’ is to artificially create a ‘stress environment’ that would activate an intra-cellular signaling pathway for secreting cytokines/chemokines and other mediators to seek the survival path.

### Analyses of circulating levels of creatinine and inflammatory biomarkers

Blood samples were stored at −80°C until analyses of tumor necrosis factor (TNF)-α and creatinine were performed in batches at the end of the experiment. Serum TNF-α concentration was assessed in duplicate with a commercially available ELISA kit (R&D Systems, Inc. Minneapolis, MN, USA). Intra-individual variability in TNF-α level was assessed in each group. The mean intra-assay coefficients of variance were all less than 4.0%. Circulating levels of creatinine and white blood cell (WBC) count were measured prior to and at 72 hours after CLP with standard laboratory methods.

### Histopathology scoring of kidney injury at 72 hours after the CLP procedure

Histopathology scoring was determined in blinded fashion as previously reported [18]. Briefly, the kidney specimens from all animals were fixed in 10% buffered formalin, embedded in paraffin, sectioned at 5 mm and stained (hematoxylin and eosin; H & E) for light microscopy. The score reflected the grading of tubular necrosis, loss of brush border, cast formation and tubular dilatation in 10 randomly chosen, non-overlapping fields (200x) for each animal as follows: 0 (none), 1 (≤10%), 2 (11% to 25%), 3 (26% to 45%), 4 (46% to 75%) and 5 (≥76%).

### Histological assessment of lung injury and crowded score for lung parenchyma

To identify alveolar sac distribution in lung parenchyma, the lung specimens from all animals were fixed in 10% buffered formalin before embedding in paraffin; tissues were sectioned at 5 μm for light microscopy. H & E staining was performed to determine the number of alveolar sacs in a blinded fashion as we previously reported [16]. Three lung sections from each rat were analyzed and three randomly selected high-power fields (HPFs; 100x) were examined in each section. The mean number per HPF for each animal was then determined by summation of all numbers divided by 9. The extent of crowded area, which was defined as the region of thickened septa in lung parenchyma associated with partial or complete collapse of alveoli on H & E-stained sections, was also performed in a blinded fashion. The following scoring system [16] was adopted: 0 = no detectable crowded area; 1 = <15% of crowded area; 2 = 15% to 25% of crowded area; 3 = 25% to 50% of crowded area; 4 = 50% to 75% of crowded area; 5 = >75% to 100% of crowded area/per HPF.

### Immunohistochemical and immunofluorescent studies

The procedures and protocols for immunohistochemical (IHC) and immunofluorescent (IF) examinations were also based on our recent study [16]. Briefly, for IHC staining, rehydrated paraffin sections were first treated with 3% H_2_O_2_ for 30 minutes and incubated with Immuno-Block reagent (BioSB, Santa Barbara, CA, USA) for 30 minutes at room temperature. Sections were then incubated with primary antibodies specifically against glutathione peroxidase (GPx; 1:500, Abcam, Cambridge, MA, USA) glutathione reductase (GR; 1:100, Abcam, Cambridge, MA, USA) and phosphorylated H2AX (γ-H2AX, Cell signaling, Danvers, MA, USA) at 4°C overnight. Irrelevant antibodies (p53 (1:500, Abcam, Cambridge, MA, USA) and mouse control immunoglobulin G (IgG, Abcam, Cambridge, MA, USA)) provided controls in the current study. IF staining was performed for the examinations of CD31 (1:200, SeroTec, Raleigh, NC, USA) using the respective primary antibody; again, irrelevant antibodies were used as controls. Three sections of lung and kidney specimens were analyzed in each rat. For quantification, three randomly selected HPFs (x200 for IHC and IF studies) were analyzed in each section. The mean number per HPF for each animal was then determined by summation of all numbers divided by 9. An IHC-based scoring system was adopted for semi-quantitative analyses of GR and GPx in kidney as a percentage of positive cells in blinded fashion (score of positively-stained cells for GR and GPx: 0 = no stain; 1 = <15%; 2 = 15% to approximately 25%; 3 = 25% to approximately 50%; 4 = 50% to approximately 75%; 5 = >75% to 100%/per HPF).

### Western blot analysis of left lung specimens

Equal amounts (10 to 30 μg) of protein extracts from the left lung were loaded and separated by SDS-PAGE using 8% to 10% acrylamide gradients. Following electrophoresis, the separated proteins were transferred electrophoretically to a polyvinylidene difluoride (PVDF) membrane (Amersham Biosciences, Piscataway, NJ, USA). Nonspecific proteins were blocked by incubating the membrane in blocking buffer (5% nonfat dry milk in T-TBS containing 0.05% Tween 20) overnight. The membranes were incubated with monoclonal antibodies against intercellular adhesion molecule (ICAM)-1 (1: 2000, Abcam, Cambridge, MA, USA), polyclonal antibodies against TNF-α (1: 1000, Cell Signaling, Danvers, MA, USA), nuclear factor (NF)-κB (1: 250, Abcam, Cambridge, MA, USA), metalloproteinase-9 (MMP-9) (1:3000, Abcam, Cambridge, MA, USA), GR (1:1000, Abcam, Cambridge, MA, USA), GPx (1:1000, Abcam, Cambridge, MA, USA), Cytochrome C (1:2000, BD, San Jose, CA, USA), NAD(P)H quinone oxidoreductase (NQO) 1 (1: 1000, Abcam, Cambridge, MA, USA), heme oxygense (HO)-1 (1: 250, Abcam, Cambridge, MA, USA), Bax (1: 1000, Abcam, Cambridge, MA, USA), caspase 3 (1: 1000, Cell Signaling), poly (ADP-ribose) polymerase (PARP) (1: 1000, Cell Signaling, Danvers, MA, USA) and Bcl-2 (1:250, Abcam, Cambridge, MA, USA). Signals were detected with horseradish peroxidase (HRP)-conjugated goat anti- mouse, goat anti-rat or goat anti-rabbit IgG.

The Oxyblot Oxidized Protein Detection Kit was purchased from Chemicon (S7150, Billerica, MA, USA). The procedure of 2,4-dinitrophenylhydrazine (DNPH) derivatization was carried out on 6 μg of protein for 15 minutes according to the manufacturer’s instructions. One-dimensional electrophoresis was carried out on 12% SDS/polyacrylamide gel after DNPH derivatization. Proteins were transferred to nitrocellulose membranes which were then incubated in the primary antibody solution (anti-DNP 1:150) for two hours, followed by incubation with the secondary antibody solution (1:300) for one hour at room temperature. The washing procedure was repeated eight times within 40 minutes.

Immunoreactive bands were visualized by enhanced chemiluminescence (ECL; Amersham Biosciences), which was then exposed to Biomax L film (Kodak). For quantification, ECL signals were digitized using Labwork software (UVP). For oxyblot protein analysis, a standard control was loaded on each gel.

### Statistical analyses

Quantitative data are expressed as mean ± SD. Statistical analysis was performed by analysis of variance (ANOVA) followed by the Bonferroni multiple-comparison *post hoc* test. All analyses were conducted using SAS statistical software for Windows version 8.2 (SAS Institute, Cary, NC, USA). A probability value <0.05 was considered statistically significant.

## Results

### Systolic blood pressure at 0 and 72 hours after CLP/SP

The SBP did not differ among the four groups at 0 hour after the CLP procedure. However, by 72 hours after the procedure, SBP was lowest in CLP + H-ADMSC, significantly lower in CLP only than in sham control (SC) and CLP + A-ADMSC, and similar between SC and CLP + A-ADMSC (Table 1).

**Table 1:** Blood pressure, hematologic and biochemical studies among the four groups

**Variables**	**Group 1**	**Group 2**	**Group 3**	**Group 4**	***P*-value**
Systolic blood pressure (mmHg)					
At 0 hour	125.8 ± 5.5	123.4 ± 4.7	119.5 ± 4.6	117.5 ± 4.2	0.647
At 72 hours	129.1 ± 7.6^a^	102.6 ± 5.6^b^	82.1 ± 5.8^c^	119. 1 ± 7.7^a^	<0.001
At 24 hours					
Creatinine	0.51 ± 0.12	0.77 ± 0.15	0.74 ± 0.25	0.66 ± 0.14	0.051
WBC count	8.1 ± 2.0	4.9 ± 2.6	7.7 ± 2.5	6.8 ± 1.4	0.057
At 72 hours					
Creatinine	0.5 ± 0.07^a^	0.78 ± 0.14^b^	0.83 ± 0.17^b^	0.61 ± 0.11^a^	<0.001
WBC count	7.7 ± 1.9^a^	12.6 ± 2.8^b^	9.2 ± 3.1^c^	9.5 ± 2.3^c^	0.016
Serum TNF-α level (ng/mL)					
At 0 hour	1.92 ± 0.81	1.89 ± 1.04	2.13 ± 1.18	2.22 ± 1.40	0.887
At 24 hours	2.16 ± 1.46^a^	11.6 ± 2.72^b^	15.9 ± 5.4^c^	12.21 ± 5.81^b^	<0.001
At 72 hours	2.4 ± 1.58^a^	5.92 ± 1.92^b^	8.53 ± 8.56^b^	4.24 ± 1.27^c^	<0.001

Data are expressed as means ± SD. Group 1, sham control; group 2, cecal ligation and puncture (CLP); group 3, CLP + healthy adipose-derived mesenchymal stem cell (H-ADMSC); group 4, CLP + adipose (A)-ADMSC. Symbols (^a^, ^b^, ^c^) indicate significant difference (at 0.05 level) by Bonferroni multiple-comparison *post hoc* test. WBC, white blood cells.

### Circulating levels of creatinine and inflammatory biomarkers after the CLP procedure

At 24 hours after the CLP procedure, the serum creatinine level and WBC count did not differ among all groups (Table 1). However, at 72 hours after the procedure, the serum creatinine level was significantly higher in CLP only and CLP + H-ADMSC than in SC and CLP + A-ADMSC, but there was no significant difference between CLP only and CLP + H-ADMSC or between SC and CLP + A-ADMSC (Table 1). In addition, the WBC count was significantly highest in CLP only, significantly higher in CLP + H-ADMSC and CLP + A-ADMSC than in SC, but it exhibited no difference between CLP + H-ADMSC and CLP + A-ADMSC at 72 hours after the procedure (Table 1).

The circulating level of TNF-α was similar among the four groups prior to the procedure, whereas it was highest in CLP + H-ADMSC, significantly higher in groups CLP only and CLP + A-ADMSC than in SC, but it displayed no difference between groups CLP only and CLP + A-ADMSC at 24 hours after the procedure (Table 1). Moreover, it was significantly higher in CLP only and CLP + H-ADMSC than SC and CLP + A-ADMSC, significantly higher in CLP + A-ADMSC than in SC, but it showed no difference between CLP only and CLP + H-ADMSC at 72 hours after the procedure (Table 1).

### Flow cytometric analysis of apoptosis and protein expressions of inflammatory biomarkers in lung and kidney at 72 hours after the CLP procedure

**Figure 1:**
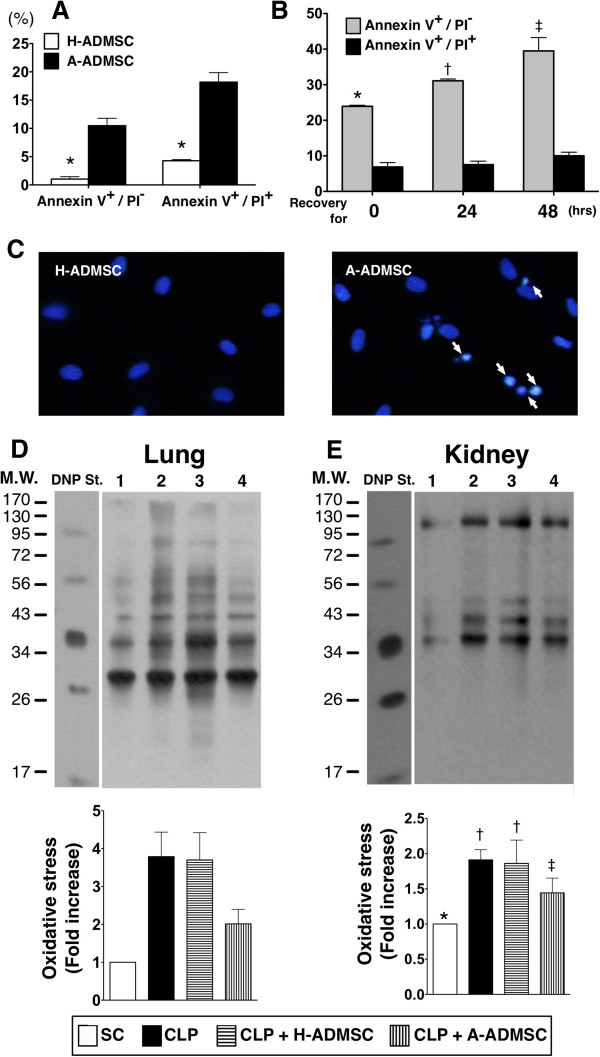
**Flow cytometric results of cellular apoptosis and Western blot results of oxidative stress. A)** Compares the incidence of early (annexin V+/PI-) and late (annexin V+/PI+) phases of apoptosis between healthy adipose-derived mesenchymal stem cells (H-ADMSC) and apoptotic **(A)** (that is, 96 hours of serum-free cell culture) ADMSC (A-ADMSC). **B)** For A-ADMSCs, early apoptosis and late apoptosis increased at 24 hours and further increased by 48 hours after re-culture (after 96 hours of serum-free cell culture) in normal culture medium with adequate nutrition. * versus H-ADMSC in early or late phase of cellular apoptosis, *P* <0.0001. **C)** DAPI stain (nuclei were stained in blue color) indicated the cell apoptosis (white arrows). Apoptosis (white arrows) in A-ADMSC was markedly increased at 48 hours after being re-cultured in normal culture medium with adequate nutrition. **D** and **E)** Oxidized protein in lung **(D)** and kidney **(E)** at 72 hours after cecal ligation and puncture (CLP) procedure. DNP, 1–3 dinitrophenylhydrazone; M.W., molecular weight (Note: The right lane and left lane shown on the upper panel represent control oxidized molecular protein standard and protein molecular weight marker, respectively). 1, sham control (SC); 2, cecal ligation and puncture (CLP); 3, CLP + H-ADMSC; 4, CLP + A-DMSC. * versus other groups with different symbols, *P* <0.001. Statistical analysis using one-way ANOVA, followed by the Bonferroni multiple comparison *post hoc* test (n = 8). Symbols (*, †, ‡) indicate significant difference (<0.05). ANOVA, analysis of variance; DAPI, 4*'*,6-diamidino-2-phenylindole.

Flow cytometric analyses demonstrated that early apoptosis (annexin V+/PI-) and late apoptosis (annexin V+/PI+) were significantly higher in the A-ADMSC group (that is, by 96 hours of serum-free cell culture) than in the H-ADMSC group (Figure 1A, 1B). Interestingly, in the A-ADMSC group, the proportion of cells in early apoptosis (annexin V+/PI-) and late apoptosis (annexin V+/PI+) was increased at 24 hours, and further increased by 48 hours after re-culture (after 96 hours of serum-free cell culture) in normal culture medium with adequate nutrition. These findings might suggest that a certain percentage of A-ADMSC would proceed to apoptosis after being transfused into the rat circulation, especially in the setting of sepsis syndrome.

By 72 hours after the three time points of ADMSC treatment (that is, 1.2 × 10^6^ of H-ADMSCs or A-ADMSCs at time points of 0.5 hour, 6 hours, and 18 hours after the CLP procedure), the expressions of oxidized protein in lung (Figure 1C, 1D) and kidney (Figure 1E, 1F), an index of oxidative stress, were significantly higher in CLP only and CLP + H-ADMSC than in SC and CLP + A-ADMSC, and significantly higher in CLP + A-ADMSC than in SC, but there was no difference between CLP only and CLP + H-ADMSC.

**Figure 2:**
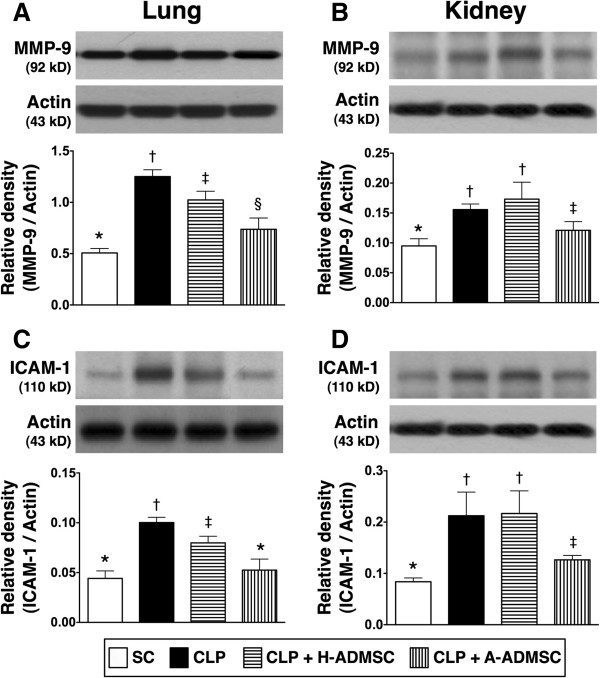
**Protein expressions of MMP-9 and ICAM-1 in lung and kidney at 72 hours after the CLP procedure. A** and **B)** The protein expression of matrix metalloproteinase (MMP)-9 in lung **(A)** and kidney **(B)**. For lung: * versus other groups with different symbols, *P* <0.001. For kidney: * versus other groups with different symbols, *P* <0.001. **C** and **D)** The protein expressions of intercellular adhesion molecule (ICAM)-1 in lung **(C)** and kidney **(D)**. For lung: * versus other groups with different symbols, *P* <0.001. For kidney: * versus other groups with different symbols, *P* <0.001. Statistical analysis using one-way ANOVA, followed by the Bonferroni multiple comparison *post hoc* test (n = 8). Symbols (*, †, ‡, §) indicate significant difference (<0.05). SC, sham control; CLP, cecal ligation and puncture; H-ADMSC, healthy adipose-derived mesenchymal stem cell; A-ADMSC, apoptotic adipose-derived mesenchymal stem cell. ANOVA, analysis of variance.

Additionally, the protein expression of MMP-9 in lung parenchyma, an index of inflammation, was highest in CLP only, significantly higher in CLP + H-ADMSC than in SC and CLP + A-ADMSC, but there was no difference between SC and CLP + A-ADMSC (Figure 2A). In addition, its expression in kidney parenchyma was significantly higher in groups CLP only and SC and CLP + H-ADMSC than in SC and CLP + A-ADMSC, and significantly higher in CLP + A-ADMSC than in SC, but it exhibited no difference between CLP only and CLP + H-ADMSC (Figure 2B).

The protein expression of ICAM-1, another inflammatory biomarker, in lung parenchyma, was significantly higher in CLP only than in the other groups, significantly higher in CLP + H-ADMSC than in groups SC and CLP + A-ADMSC, but it did not differ between SC and CLP + A-ADMSC (Figure 2C). Accordingly, its expression in kidney parenchyma showed a similar pattern to that of oxidized protein expression among the four groups (Figure 2D).

**Figure 3:**
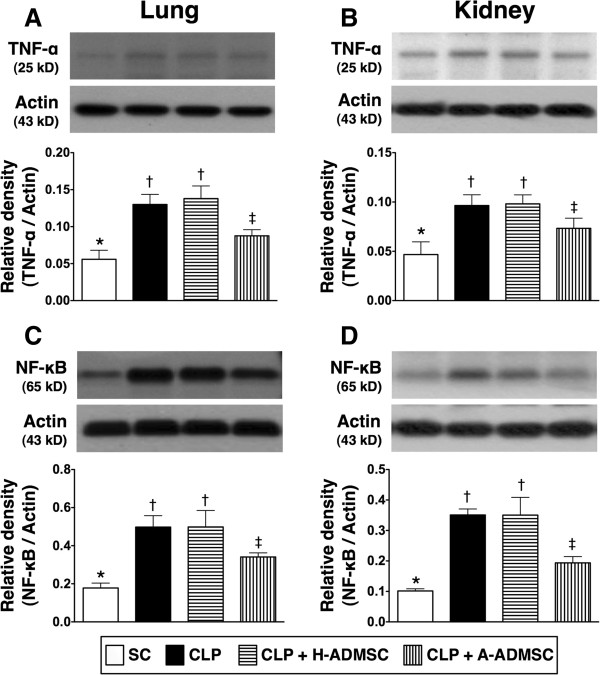
**Protein expression of TNF-α and NF-κB in lung and kidney at 72 hours after the CLP procedure. A** and **B)** The protein expression of tumor necrosis factor alpha (TNF-α) in lung **(A)** and kidney **(B)**. For lung: * versus other groups with different symbols, *P* <0.001. For kidney: * versus other groups with different symbols, *P* <0.001. **C** and **D)** The protein expression of nuclear factor (NF)- κB in lung **(C)** and kidney **(D)**. For lung: * versus other groups with different symbols, P <0.001. For kidney: * versus other groups with different symbols, *P* <0.0001. Statistical analysis using one-way ANOVA, followed by the Bonferroni multiple comparison *post hoc* test (n = 8). Symbols (*, †, ‡, §) indicate significant difference (<0.05). SC, sham control; CLP, cecal ligation and puncture; H-ADMSC, healthy adipose-derived mesenchymal stem cell; A-ADMSC, apoptotic adipose-derived mesenchymal stem cell. ANOVA, analysis of variance.

Moreover, the protein expressions of TNF-α and NF-κB, two inflammatory biomarkers in lung and kidney parenchyma, were highest in CLP + H-ADMSC, lowest in SC, significantly higher in CLP only than in CLP + A-ADMSC (Figure 3A, 3B, 3C, 3D).

### The protein expressions of apoptotic and anti-apoptotic biomarkers at 72 hours after the CLP procedure

**Figure 4:**
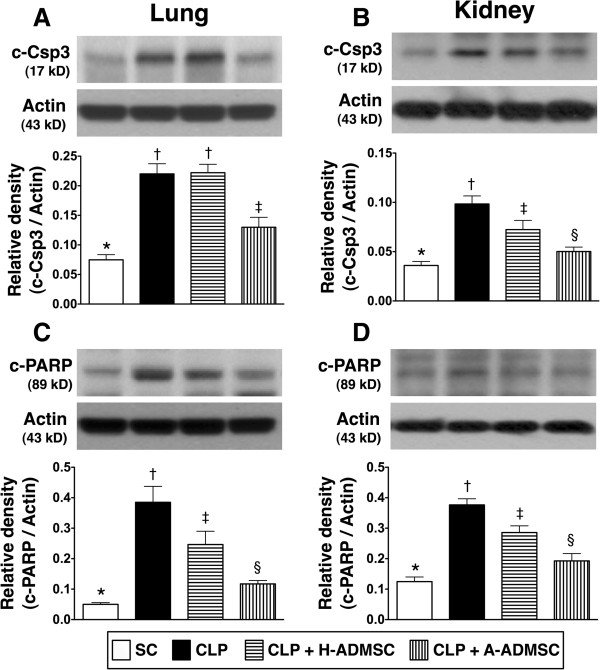
**The protein expressions of apoptotic biomarkers in lung and kidney at 72 hours after the CLP procedure. A** and **B)** The protein expression of cleaved caspase 3 (C-Csp3) in lung **(A)** and kidney **(B)**. For lung: * versus other groups with different symbols, *P* <0.001. For kidney: * versus other groups with different symbols, *P* <0.001. **C** and **D)** The protein expression of cleaved poly (ADP-ribose) polymerase (C-PARP) in lung **(C)** and kidney **(D)**. For lung: * versus other groups with different symbols, *P* <0.0001. For kidney: * versus other groups with different symbols, *P* <0.001. Statistical analysis using one-way ANOVA, followed by the Bonferroni multiple comparison *post hoc* test (n = 8). Symbols (*, †, ‡, §) indicate significant difference (<0.05). SC, sham control; CLP, cecal ligation and puncture; H-ADMSC, healthy adipose-derived mesenchymal stem cell; A-ADMSC, apoptotic adipose-derived mesenchymal stem cell. ANOVA, analysis of variance.

The protein expression of cleaved (that is, active form) caspase 3 in lung and kidney, an index of apoptosis, was significantly higher in CLP only and CLP + H-ADMSC than in SC and CLP + A-ADMSC, and significantly higher in CLP + A-ADMSC than in SC (Figure 4-A, 4-B). In addition, this parameter in kidney was significantly higher in CLP only than in CLP + H-ADMSC, whereas there was no difference in lung parenchyma between CLP only and CLP + H-ADMSC (Figure 4A, 4B). Furthermore, the protein expression of PARP in lung (4-C) and kidney (4-D), the substrate of caspase 3, was highest in CLP only, lowest in SC, and significantly higher in CLP + H-ADMSC than in CLP + A-ADMSC.

**Figure 5:**
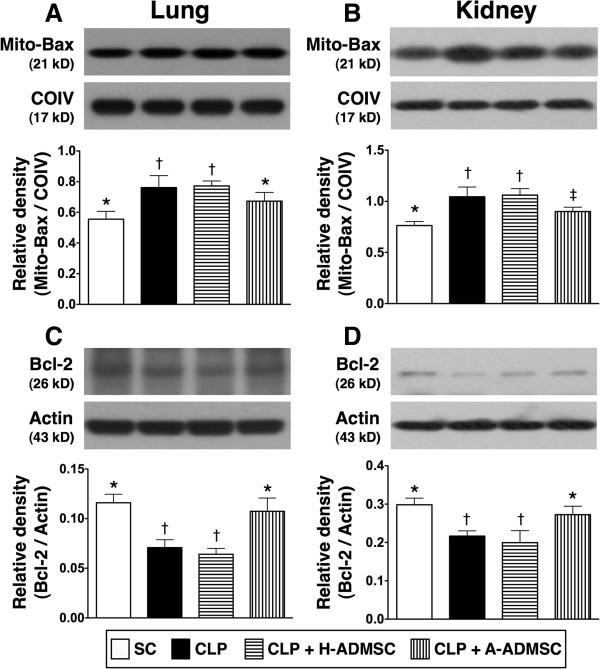
**The protein expressions of apoptotic and anti-apoptotic biomarkers in lung and kidney at 72 hours after the CLP procedure. A** and **B)** The protein expression of mitochondrial (Mito) Bax in lung **(A)** and kidney **(B)**. For lung: * versus other groups with different symbols, *P* <0.01. For kidney: * versus other groups with different symbols, *P* <0.01. **C** and **D)** The protein expression of Bcl-2 in lung **(C)** and kidney **(D)**. For lung: * versus other groups with different symbols, *P* <0.001. For kidney: * versus other groups with different symbols, *P* <0.01. Statistical analysis using one-way ANOVA, followed by the Bonferroni multiple comparison *post hoc* test (n = 8). Symbols (*, †, ‡) indicate significant difference (<0.05). SC, sham control; CLP, cecal ligation and puncture; H-ADMSC, healthy adipose-derived mesenchymal stem cell; A-ADMSC, apoptotic adipose-derived mesenchymal stem cell. ANOVA, analysis of variance.

The mitochondrial Bax protein expression in lung and kidney parenchyma, an indicator of apoptosis, was significantly higher in CLP only and CLP + H-ADMSC than in SC and CLP + A-ADMSC (Figure 5A, 5B). Additionally, this parameter was significantly higher in CLP + A-ADMSC than in SC in kidney parenchyma, but it exhibited no difference between SC and CLP + A-ADMSC in lung parenchyma (Figure 5A, 5B). On the other hand, the protein expression of Bcl-2, an indicator of anti-apoptosis, was significantly lower in CLP only and CLP + H-ADMSC than in SC and CLP + A-ADMSC, but it did not differ between CLP only and CLP + H-ADMSC or between SC and CLP + A-ADMSC (Figure 5C, 5D).

### The protein expressions of anti-oxidant biomarkers at 72 hours after the CLP procedure

**Figure 6:**
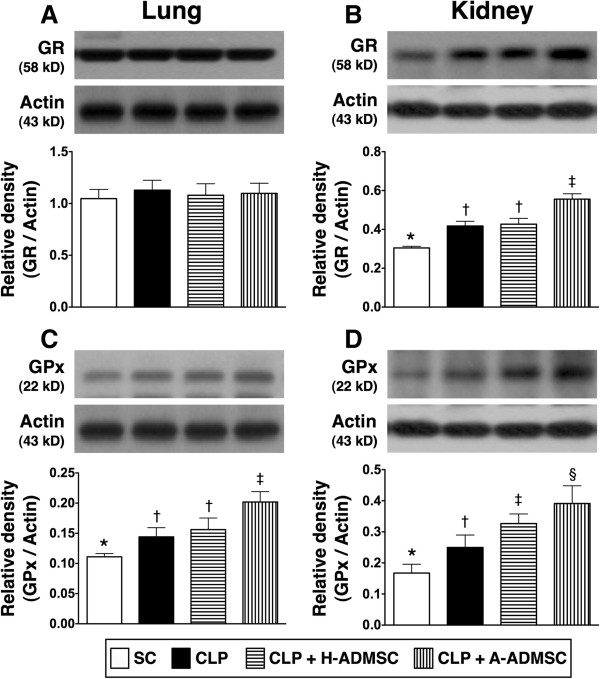
**The protein expression of GR and GPx in lung and kidney at 72 hours after the CLP procedure. A** and **B)** The protein expression of glutathione reductase (GR) in lung **(A)** and kidney **(B)**. For lung: * versus other groups with different symbols, *P* >0.1. For kidney: * versus other groups with different symbols, *P* <0.001. **C** and **D)** The protein expression of glutathione peroxidase (GPx) in lung **(C)** and kidney **(D)**. For lung: * versus other groups with different symbols, *P* <0.008. For kidney: * versus other groups with different symbols, *P* <0.0001. Statistical analysis using one-way ANOVA, followed by the Bonferroni multiple comparison *post hoc* test (n = 8). Symbols (*, †, ‡, §) indicate significant difference (<0.05). SC, sham control; CLP, cecal ligation and puncture; H-ADMSC, healthy adipose-derived mesenchymal stem cell; A-ADMSC, apoptotic adipose-derived mesenchymal stem cell. ANOVA, analysis of variance.

The protein expression of GR in the lung, an indicator of anti-oxidation, did not differ among the four groups (Figure 6A). However, this protein expression in the kidney was lowest in SC and highest in CLP + A-ADMSC, but there was no significant difference between CLP only and CLP + H-ADMSC (Figure 6B). Additionally, the protein expression of GPx in the lung was highest in CLP + A-ADMSC, significantly higher in CLP only and CLP + H-ADMSC than in SC, but there was no difference between CLP only and CLP + H-ADMSC (Figure 6C). Furthermore, its expression in the kidney was lowest in SC, highest in CLP + A-ADMSC, significantly higher in CLP + H-ADMSC than in CLP only (Figure 6D).

**Figure 7:**
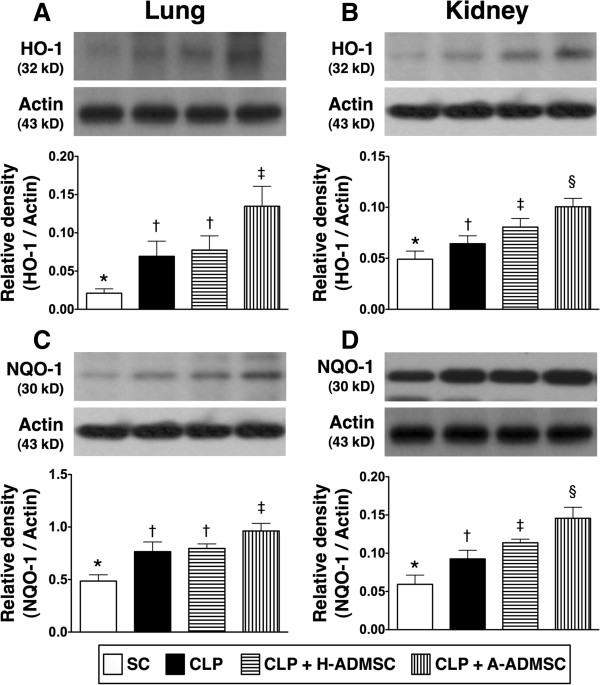
**The protein expression of HO-1 and NQO 1 in lung and kidney at 72 hours after the CLP procedure. A** and **B)** The protein expression of heme oxygense (HO)-1 in lung **(A)** and kidney **(B)**. For lung: * versus other groups with different symbols, *P* <0.0001. For kidney: * versus other groups with different symbols, *P* <0.001. **C** and **D)** The protein expression of NAD(P)H quinone oxidoreductase (NQO) 1 in lung **(C)** and kidney **(D)**. For lung: * versus other groups with different symbols, *P* <0.001. For kidney: * versus other groups with different symbols, *P* <0.001. Statistical analysis using one-way ANOVA, followed by the Bonferroni multiple comparison *post hoc* test (n = 8). Symbols (*, †, ‡, §) indicate significant difference (<0.05). SC, sham control; CLP, cecal ligation and puncture; H-ADMSC, healthy adipose-derived mesenchymal stem cell; A-ADMSC, apoptotic adipose-derived mesenchymal stem cell. ANOVA, analysis of variance.

The protein expressions of HO-1 (7-A) and NQO 1 (7-C) in lung, another two indices of anti-oxidation, were significantly higher in CLP + A-ADMSC than in other groups, significantly higher in CLP only and CLP + H-ADMSC than in SC, but there was no notable difference between CLP only and CLP + H-ADMSC. Moreover, the expressions of these two parameters in kidney were lowest in SC, highest in CLP + A-ADMSC, and significantly higher in CLP + H-ADMSC than in CLP only (Figure 7B, 7D).

### The protein expressions of markers of mitochondrial integrity and DNA damage at 72 h after CLP procedure

**Figure 8:**
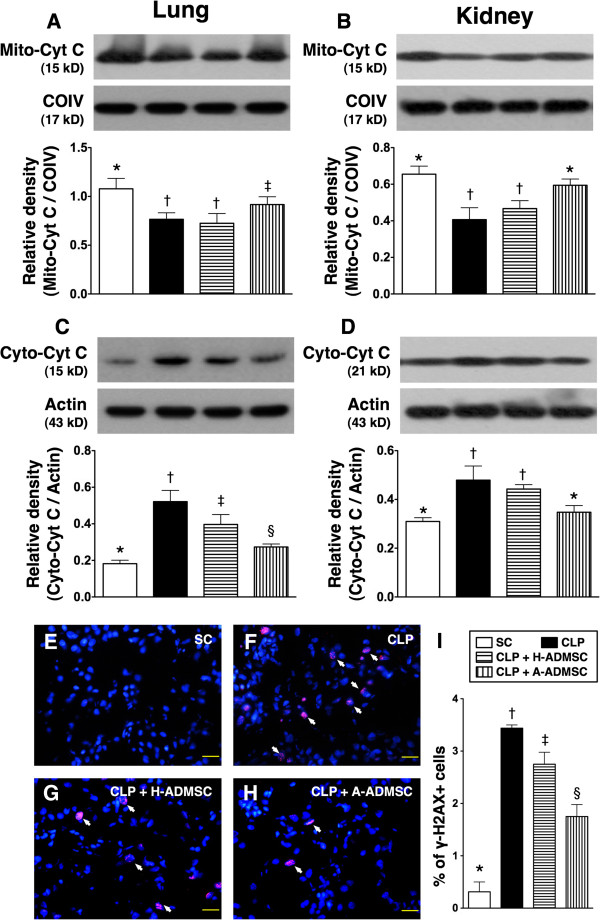
**The protein expression of mitochondrial integrity in both lung and kidney and DNA damage marker in kidney at 72 hours after the CLP procedure. A** and **B)** The protein expression of mitochondrial cytochrome C (Mito-Cyt C) in lung **(A)** and kidney **(B)**. For lung: * versus other groups with different symbols, *P* <0.01. For kidney: * versus other groups with different symbols, *P* <0.001. **C** and **D)** The protein expression of mitochondrial cytochrome C (Cyto-Cyt C) in lung **(C)** and kidney **(D)**. For lung: * versus other groups with different symbols, *P* <0.001. For kidney: * versus other groups with different symbols, *P* < 0.01. **E** to **H)** Immunofluorescent stain (400x) for γ-H2AX + cells (white arrows) in kidney. **I)** Quantitative analysis of γ-H2AX + cells (%), *P* <0.0001). DAPI stain for identification of nuclei (blue color). Scale bars in the right lower corner represent 20 μm. Statistical analysis using one-way ANOVA, followed by the Bonferroni multiple comparison *post hoc* test (n = 8). Symbols (*, †, ‡, §) indicate significant difference (<0.05). SC, sham control; CLP, cecal ligation and puncture; H-ADMSC, healthy adipose-derived mesenchymal stem cell; A-ADMSC, apoptotic adipose-derived mesenchymal stem cell. ANOVA, analysis of variance; DAPI, 4*'*,6-diamidino-2-phenylindole.

Protein expression of mitochondrial cytochrome C, an indicator of mitochondrial integrity in lung, was significantly higher in SC and CLP + A-ADMSC than in CLP only and CLP + H-ADMSC, and significantly higher in SC than in CLP + A-ADMSC, but it showed no difference between CLP only and CLP + H-ADMSC (Figure 8A). Conversely, the cytosolic expression of this biomarker, an index of mitochondrial damage, was lowest in SC and highest in CLP only, significantly higher in CLP + H-ADMSC than in CLP + A-ADMSC (Figure 8C). Similarly, the mitochondrial level of this biomarker in kidney was significantly higher in SC and CLP + A-ADMSC than in CLP only and CLP + H-ADMSC, but there was no difference between SC and CLP + A-ADMSC or between CLP only and CLP + H-ADMSC (Figure 8B). Furthermore, the cytosolic level of this biomarker in kidney showed an opposite pattern of mitochondrial level among the four groups (Figure 8D).

IF staining of kidney parenchyma showed that the population of γ-H2AX (+) cells, an indicator of DNA damage, was highest in CLP only, lowest in SC and significantly higher in CLP + H-ADMSC than in CLP + A-ADMSC (Figure 8E to 8I).

### Histopathology of the kidney at 72 hours after the CLP procedure

**Figure 9:**
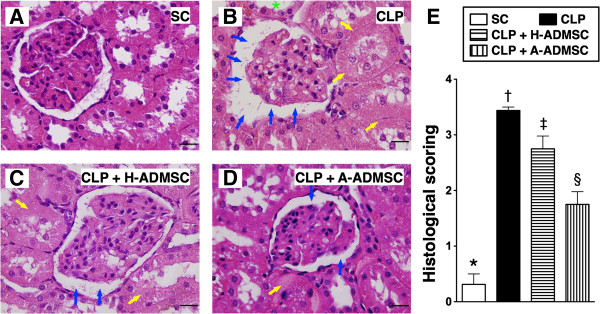
**Histopathological scoring of kidney injury at 72 hours after the CLP procedure. A** to **D)** H & E stain (400x) showing a significantly higher degree of loss of brush border in renal tubules (yellow arrows), tubular necrosis (green asterisk) and dilatation of Bowman’s capsule (blue arrows) in the CLP group than in the other groups. **E)** * versus other groups with different symbols (*, †, ‡, §), *P* <0.0001. All statistical analyses using one-way ANOVA, followed by the Bonferroni multiple comparison *post hoc* test (n = 8). Symbols (*, †, ‡, §) indicate significance (at 0.05 level). Scale bars in the right lower corner represent 20 μm. SC, sham control; CLP, cecal ligation and puncture; H-ADMSC, healthy adipose-derived mesenchymal stem cell; A-ADMSC, apoptotic adipose-derived mesenchymal stem cell. ANOVA, analysis of variance.

To determine the effects of H-ADMSC and A-ADMSC treatment on the severity of CLP-induced renal injury, a histological scoring system based on the typical microscopic features of acute tubular damage (including extensive tubular necrosis and dilatation, cast formation, and loss of brush border) was adopted (Figure 9). This injury score was highest in CLP only, significantly higher in CLP + H-ADMSC than in SC and CLP + A-ADMSC, and significantly higher in CLP + A-ADMSC than in SC.

### The expressions of anti-oxidative activity in the kidney at 72 hours after the CLP procedure

**Figure 10:**
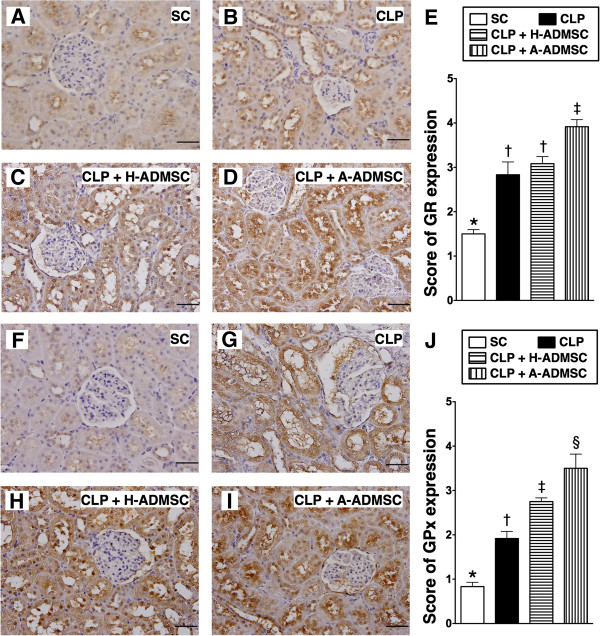
**Immunohistochemical (IHC) staining for kidney expressions of anti-oxidative markers at 72 hours after the CLP procedure. A** to **D)** Microscopic findings of IHC stain (200x) for glutathione reductase (GR)-positive cells (brown) in renal parenchyma of the four groups. Scale bars in the right lower corner represent 50 μm. **E)** * versus other groups with different symbols (*, †, ‡), *P* <0.0001. **F** to **I)** Microscopic findings of IHC stain (200x) for glutathione peroxidase (GPx)-positive cells (brown) in renal parenchyma of the four groups. Scale bars in the right lower corner represent 50 μm. **J)** * versus other groups with different symbols (*, †, ‡, ¶), *P* <0.0001. All statistical analyses using one-way ANOVA, followed by the Bonferroni multiple comparison *post hoc* test (n = 8). Symbols (*, †, ‡, §) indicate significance (at 0.05 level). SC ,sham control; CLP, cecal ligation and puncture; H-ADMSC, healthy adipose-derived mesenchymal stem cell; A-ADMSC, apoptotic adipose-derived mesenchymal stem cell. ANOVA, analysis of variance.

IHC staining demonstrated that the expressions of GR (Figure 10A to 10E) and GPx (Figure 10F to 10J), two oxidoreductase enzymes, were highest in CLP + A-ADMSC and lowest in SC, and that of GPx was significantly higher in CLP + H-ADMSC than in CLP only, but that of GR did not differ between CLP only and CLP + H-ADMSC.

### Immunohistochemical (IHC) staining of alveolar sacs, crowded score and population of inflammatory cells in the lung parenchyma at 72 hours after the CLP procedure

**Figure 11:**
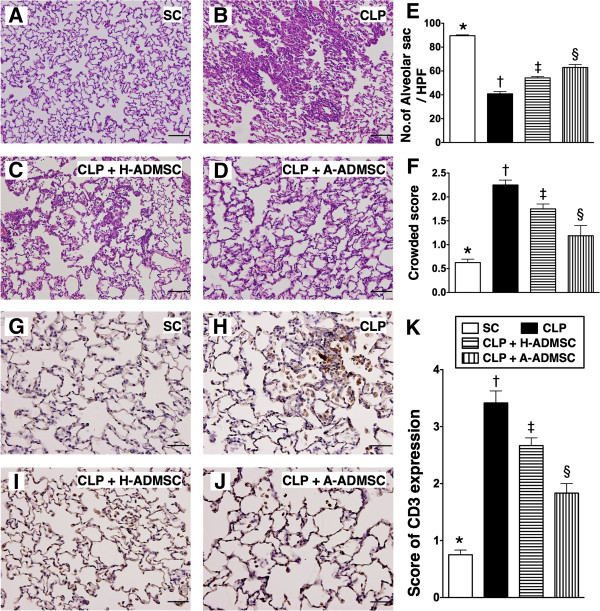
**Histopathological changes and inflammatory cell infiltration in lung parenchyma at 72 hours after the CLP procedure. A** to **D)** Microscopic findings of H & E stain (100x) of lung parencyma at 72 hours after the procedure. Scale bars in the right lower corners represent 100 μm. **E)** Quantitative analysis of the number of alveolar sacs. * versus other groups with different symbols (*, †, ‡, §), *P* <0.0001. **F)** Quantitative analysis of crowded score. * versus other groups with different symbols (*, †, ‡, §), *P* <0.001. **G** to **J)** Immunohistochemical stain (200x) for CD3+ cells in lung parencyma at 72 hours after the procedure. Scale bars in right lower corners represent 50 μm. **K)** Quantitative analysis of CD3+ cells. * versus other groups with different symbols (*, †, ‡, §), *P* <0.0001. All statistical analyses using one-way ANOVA, followed by the Bonferroni multiple comparison *post hoc* test (n = 8). Symbols (*, †, ‡, §) indicate significance (at 0.05 level). SC, sham control; CLP, cecal ligation and puncture; H-ADMSC, healthy adipose-derived mesenchymal stem cell; A-ADMSC, apoptotic adipose-derived mesenchymal stem cell. ANOVA, analysis of variance.

IHC staining showed that the number of alveolar sacs was lowest in the CLP only group, significantly lower in CLP + H-ADMSC than in SC and CLP + A-ADMSC and significantly lower in CLP + A-ADMSC than in SC (Figure 11A to 11E). Microscopically, the lung parenchyma was most crowded in CLP only, significantly more crowded in CLP + H-ADMSC than in SC and CLP + A-ADMSC, and significantly more crowded in CLP + A-ADMSC than in SC (Figure 11A to 11D, 11F).

IHC staining for the number of CD3+ cells, an index of inflammatory cells in lung parenchyma, was highest in CLP only and lowest in SC, significantly higher in CLP + H-ADMSC than in CLP + A-ADMSC (Figure 11G to 11K).

### The protein expressions of inflammatory biomarkers in the lung and kidney at 72 hours after the CLP procedure after treatment with one dose of ADMSC

**Figure 12:**
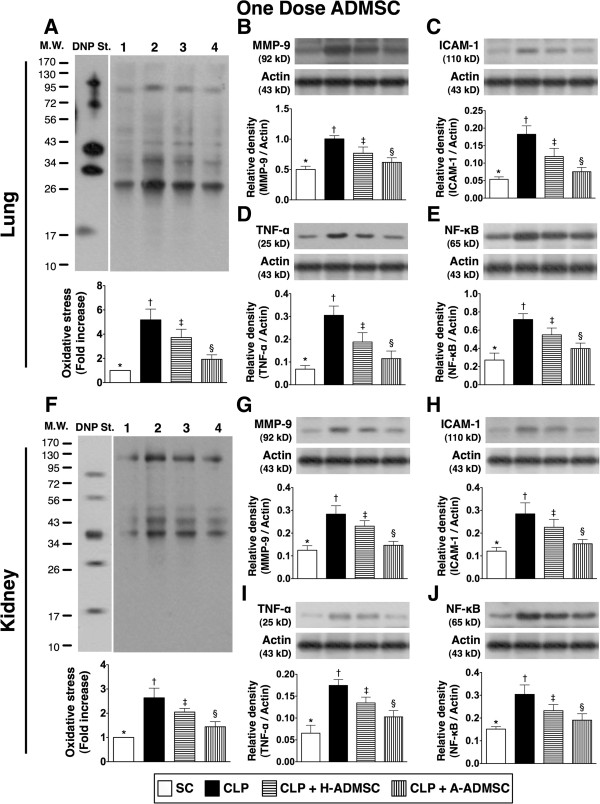
**The protein expressions of oxidative stress and inflammatory biomarkers in both lung and kidney at 72 hours after the CLP procedure with one dose of ADMSC treatment.** The protein expressions of oxidized protein **(A, F)**, matrix metalloproteinase (MMP)-9 **(B, G)**, intercellular adhesion molecule (ICAM) **(C, H)**, tumor necrosis factor alpha (TNF-α) **(D, I)**, and nuclear factor (NF)- κB **(E, J)** in lung and kidney were highest in CLP and lowest in SC, and significantly higher in CLP + H-ADMSC than in CLP + A-ADMSC (all *P* values <0.005). * versus other groups with different symbols (*, †, ‡, §), *P* <0.005. DNP, 1–3 dinitrophenylhydrazone; M.W., molecular weight (Note: Right lane and left lane shown on the upper panel represent control oxidized molecular protein standard and protein molecular weight marker, respectively). 1, sham control (SC); 2, cecal ligation and puncture (CLP); 3, CLP + H-ADMSC; 4, CLP + A-ADMSC. All statistical analyses using one-way ANOVA, followed by the Bonferroni multiple comparison *post hoc* test (n = 6). Symbols (*, †, ‡, §) indicate significance (at 0.05 level). SC, sham control; CLP, cecal ligation and puncture; H-ADMSC, healthy adipose-derived mesenchymal stem cell; A-ADMSC, apoptotic adipose-derived mesenchymal stem cell. ANOVA, analysis of variance.

Interestingly, one recent study has shown that, as compared to a lower dose of ADMSC, the higher dose of ADMSC did not offer an additional benefit on attenuating lung injury in the sepsis syndrome setting [29]. In addition, the results of the resent study demonstrated that treatment with three doses of H-ADMSC was inconsistent in suppressing the inflammatory mediators. Accordingly, in order to elucidate whether one dose of H-ADMSC (that is, 1.2 × 10^6^ cells) was more suitable than three doses of H-ADMSC for reducing inflammatory biomarkers in lung and kidney parenchyma, an additional twenty four SD rats were equally distributed into SC, CLP only, H-ADMSC and A-ADMSC groups and received one low dose (1.2 × 10^6^ cells) of H-ADMSCs or A-ADMSCs at 30 minutes after the CLP procedure, respectively. Interestingly, the protein expressions of oxidative stress, MMP-9, VCAM-1, TNF-α and NF-κB, in lung and kidney parenchyma were highest in CLP only, lowest in SC, and significantly higher in CLP + H-ADMSC than in CLP + A-ADMSC at 72 hours after the CLP procedure (Figure 12A to 12J). Our findings imply that one dose of H-ADMSC was better and more consistent than three time-points of strategic management (that is, high dosage of ADMSCs) for reducing the sepsis-induced kidney and lung injury.

### The immunofluorescent and immunohistochemical staining of lung and kidney at 72 hours after the CLP procedure after treatment with one dose of ADMSC

**Figure 13:**
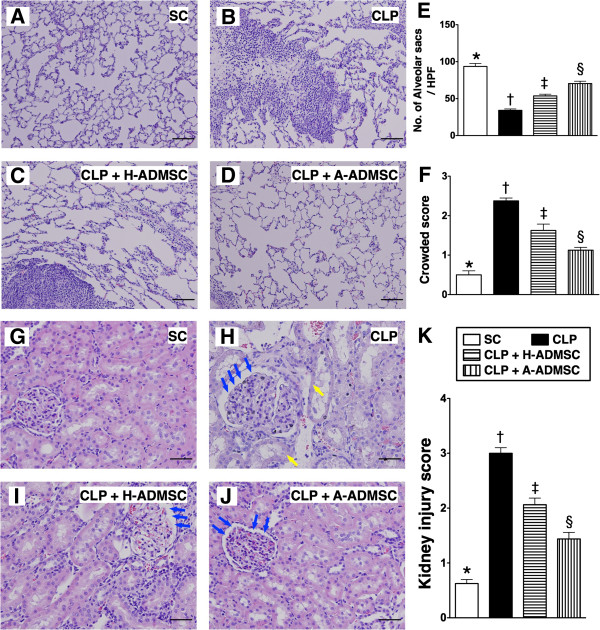
**Histopathological changes in lung and kidney at 72 hours after the CLP procedure with one dose of ADMSC treatment. A** to **D)** Microscopic findings of H & E stain (100x) of lung parenchyma at 72 hours after the procedure. Scale bars in the right lower corners represent 100 μm. **E)** Quantitative analysis of the number of alveolar sacs. * versus other groups with different symbols (*, †, ‡, §), *P* <0.001. **F)** Quantitative analysis of crowded score. * versus other groups with different symbols (*, †, ‡, §), *P* <0.001. **G** to **J)** H & E stain (200x) showing the pathological findings in kidney at 72 hours after the CLP procedure. Scale bars in the right lower corners represent 50 μm. yellow arrows = loss of brush border in renal tubules; blue arrows = dilatation of Bowman’s capsule. **K)** * versus other groups with different symbols (*, †, ‡, §), *P* <0.001. All statistical analyses using one-way ANOVA, followed by the Bonferroni multiple comparison *post hoc* test (n = 6). Symbols (*, †, ‡, §) indicate significance (at 0.05 level). SC, sham control; CLP, cecal ligation and puncture; H-ADMSC, healthy adipose-derived mesenchymal stem cell; A-ADMSC ,apoptotic adipose-derived mesenchymal stem cell. ANOVA, analysis of variance.

**Figure 14:**
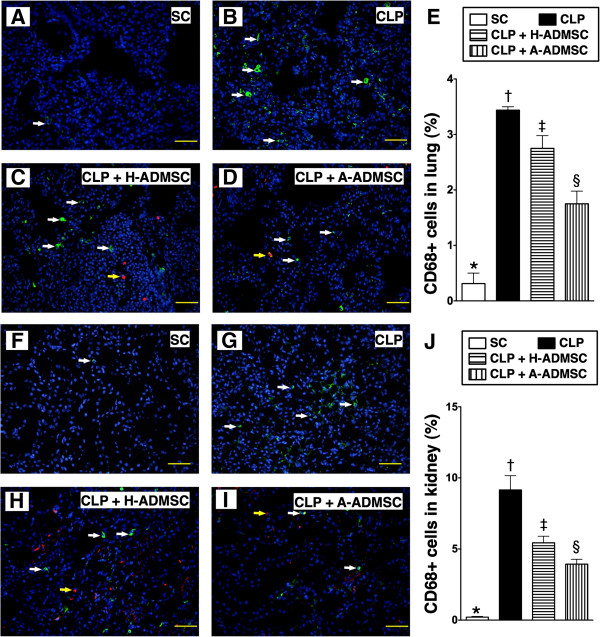
**Immunofluorescent staining for identifying infiltration of CD68+ cells in lung and kidney at 72 hours after the CLP procedure with one dose of ADMSC treatment. A** to **D)** and **F** to **I)** IF microscopic findings (200x) of the number of CD68+ cells (white arrows indicate CD68+ cells) in lung and kidney parenchyma, respectively. Scale bars in the right lower corners represent 50 μm. **E)** and **J)** Quantitative analysis of the number of CD68+ cells in lung and kidney, respectively. Yellow arrows indicated the ADMSCs. * versus other groups with different symbols (*, †, ‡, §), *P* <0.0001. All statistical analyses using one-way ANOVA, followed by the Bonferroni multiple comparison *post hoc* test (n = 6). Symbols (*, †, ‡, §) indicate significance (at 0.05 level). SC, sham control; CLP, cecal ligation and puncture; H-ADMSC, healthy adipose-derived mesenchymal stem cell; A-ADMSC, apoptotic adipose-derived mesenchymal stem cell. ANOVA, analysis of variance.

The IHC staining showed that the number of alveolar sacs in lung parenchyma was highest in SC and lowest in CLP only, and significantly lower in CLP + H-ADMSC than in CLP + A-ADMSC at 72 hours after the CLP procedure (Figure 13A to 13F) after one dose of ADMSC treatment. Conversely, the crowded score of lung parenchyma (Figure 13A to 13F) and the kidney injury score (Figure 13G to 13J) were lowest in SC and highest in CLP only, and significantly higher in CLP + H-ADMSC than in CLP + A-ADMSC at 72 hours after the CLP procedure. Additionally, the IF staining revealed that the infiltration of CD68+ cells, an index of inflammatory cells in lung (Figure 14A to 14E) and kidney (14-F to 14-J) parenchyma, displayed a pattern identical to the kidney injury score at 72 hours after the CLP procedure. These findings indicate that one dose of both H-ADMSC and A-ADMSC significantly effectively attenuated sepsis-induced lung and kidney parenchymal injury and inflammatory response, although it was less effective in H-ADMSC as compared with A-ADMSC.

## Discussion

This study, which investigated the damage caused by the CLP-induced sepsis syndrome in the lung and kidney and the therapeutic impact of ADMSC, produced several striking implications. First, our experimental study demonstrated that ALI and AKI caused by damage from CLP-induced sepsis syndrome were inevitable. Second, A-ADMSC was superior to H-ADMSC in reducing sepsis-induced ALI and AKI. Third, as compared with H-ADMSC, A-ADMSC treatment was not only more effective, but also more consistent in reducing inflammation, oxidative stress, apoptosis and histopathological perturbations in the lungs and kidneys.

Laboratory findings in the present study revealed abnormally elevated circulating levels of creatinine, TNF-α and white blood cell count as well as notably decreased SBP following CLP-induced sepsis syndrome. Undoubtedly, these experimental findings are consistent with those in the clinical setting of severe sepsis.

The principal finding in the present study is the histopathological picture of substantially aggravated pulmonary (that is, increased crowded score and reduced number of alveolar sacs) and renal (increased damage scores and γ-H2AX-positively stained cells) parenchymal damage caused by CLP-induced sepsis syndrome. Previous studies have demonstrated that ALI and AKI commonly encountered in critically ill patients [10] with sepsis syndrome were strongly associated with poor prognostic outcomes [6,9-15]. Our findings, therefore, strengthen those of the previous studies [6,9-15].

Experimental studies have revealed that stem cell treatment significantly reduced sepsis-induced organ/tissue damage through attenuating the inflammatory response and the generation of oxidative stress which, in turn, improved the prognostic outcome [23-25]. Moreover, growing evidence has shown that apoptotic/dying stem cells possess a distinctive capacity of immunomodulation [21]. Furthermore, our recent study [22] has demonstrated that, as compared with H-ADMSC + melatonin, A-ADMSC + melatonin was more effective in reducing acute IR lung injury. Surprisingly, whether A-ADMSC is superior to H-ADMSC in protection against sepsis syndrome-induced lung and kidney injuries has not been investigated. The most important finding in the present study is that treatment with A-ADMSC was found to be more effective than that with H-ADMSC in suppressing the levels of inflammation, apoptosis and oxidative stress as well as in enhancing anti-oxidation and anti-apoptosis in a rodent model of sepsis syndrome. The findings may account for the enhanced effectiveness of treatment with A-ADMSC compared to that with H-ADMSC in attenuating lung and kidney parenchymal injury in this experimental setting. Taken together, our results, in addition to reinforcing those of our recent study [22], also extend the findings of previous studies [21,23-25] and highlight the therapeutic potential of A-ADMSC in patients with severe sepsis/septic shock unresponsive to traditional antibiotic treatment.

Consistently, several of our recent studies have shown the positive therapeutic impact of H-ADMSC treatment on acute IR-induced organ injury [16,18,22]. Unexpectedly, in the current study (that is, a setting of CLP-induced sepsis syndrome), treatment at three-time points with H-ADMSC (total 3.6 × 10^6^ cells, that is, a relatively high dosage), but not A-ADMSC, showed inconsistent therapeutic actions against inflammation, apoptosis and oxidative stress, as well as in enhancing the generation of anti-apoptotic and anti-oxidant biomarkers in both pulmonary and renal parenchyma. However, as compared with untreated CLP, one low-dose H-ADMSC treatment (that is, 1.2 × 10^6^ cell administration at 30 minutes after CLP procedure) was found to reduce significantly the expressions of inflammatory biomarkers and protect the lung and kidney parenchyma from sepsis syndrome-induced injuries. Our findings are, therefore, consistent with those of the previous studies [16,18,22]. On the other hand, contrary to the anti-inflammatory effect of A-ADMSCs, the administration of three high-doses of H-ADMSCs caused paradoxical up-regulation in the expressions of inflammatory markers in the present study that cannot be satisfactorily explained. We propose three possible reasons that could explain this phenomenon. First, we suggest that in host response, particularly that which participates in the clearance of the infectious agents/products, three doses of H-ADMSC, especially the third dosage, might elicit a hyper-reactive immune response that mimics delayed hypersensitivity rather than immune desensitization. Second, it may be that the dose of H-ADMSC used in the study was just too high, and 30% of the apoptotic cells just served to reduce the dose of live cells to a better point on the dose–response curve. Therefore, our additional studies, that is, with the lower dose, only serve to give credence to this alternative explanation. Interestingly, one recent study has demonstrated that an elevated dose of ADMSC did not contribute to enhanced protection against lung injury in the setting of sepsis syndrome [29]. Of importance is the fact that an overdose of cell treatment might even be harmful to the lung parenchyma [29]. The results of that study [29] may, therefore, at least in part, explain the findings of our study. Third, perhaps another more convincing explanation is that the ‘stress’ of serum deprivation served to prime the surviving cells rather than that 30% of the stressed cell apoptosis in our CLP model, making them more effective to protect against organ damage.

This study has limitations. First, it was not designed to investigate the long-term therapeutic effect of ADMSC treatment against sepsis-induce pulmonary and renal parenchymal damage. Second, this study did not compare the therapeutic effect of a single versus multiple doses of ADMSC on outcomes after CLP-induced sepsis syndrome. Third, this study did not induce 100% of the A-ADMSCs for the treatment of sepsis syndrome. Therefore, we do not know whether 100% of A-ADMSC is more effective than the regimen of the present study for treating sepsis syndrome.

## Conclusions

Treatment with A-ADMSC was superior to that with H-ADMSC in attenuating sepsis syndrome-induced lung and kidney parenchymal injury through suppressing inflammation, apoptosis and oxidative stress as well as enhancing anti-oxidation and anti-apoptosis in a rodent model. The optimal dose of ADMSC for the treatment of different disease entities should be clarified in pre-clinical study prior to its clinical application.

## Abbreviations

ADMSC: apoptotic adipose-derived mesenchymal stem cell; AKI: acute kidney injury; ALI: acute lung injury; ANOVA: analysis of variance; CLP: cecal ligation puncture; (D)MEM: (Dulbecco’s) modified Eagle’s medium; DNPH: 2,4-dinitrophenylhydrazine; ECL: enhanced chemiluminescence; ELISA: enzyme-linked immunosorbent assay; GPx: glutathione peroxidase; GR: glutathione reductase; H & E: hematoxylin and eosin; HO-1: heme oxygenase-1; HPF: high-power field; H2AX: H2A histone family, member X; ICAM-1: intercellular adhesion molecule-1; IR: ischemia-reperfusion; MMP-9: matrix metalloproteinase-9; NF-κB: nuclear factor kappa-light-chain-enhancer of activated B cells; NQO-1: NAD(P)H:quinone oxidoreductase-1; PARP: poly (ADP-ribose) polymerase; PI: propidium iodide; PVDF: polyvinylidene difluoride; TNF-α: tumor necrosis factor-α; SBP: systolic blood pressure; WBC: white blood cell.

## Competing interests

The authors declare that they have no competing interests.

## Authors’ contributions

PHS and CLC participated in the design of the study, data acquisition and analysis as well as drafting the manuscript. THT, LTC, YLC, and CCY were responsible for the laboratory assays and troubleshooting. SC, KHY, HTC, and HWC (Chang) participated in data acquisition, analysis and interpretation. SL, HHC (Chen), and HKY conceived of the study, and participated in its design and coordination and helped in drafting the manuscript. All authors read and approved the final manuscript.
